# Protocol for the Brain Health Support Program Study of the Canadian Therapeutic Platform Trial for Multidomain Interventions to Prevent Dementia (CAN-THUMBS UP): A Prospective 12-Month Intervention Study

**DOI:** 10.14283/jpad.2023.65

**Published:** 2023-06-15

**Authors:** Howard H. Feldman, S. Belleville, H. B. Nygaard, M. Montero-Odasso, J. Durant, J.-L. Lupo, C. Revta, S. Chan, M. Cuesta, P. J. Slack, S. Winer, P. W. H. Brewster, S. M. Hofer, A. Lim, A. Centen, D. M. Jacobs, N. D. Anderson, J. D. Walker, M. R. Speechley, G. Y. Zou, H. Chertkow, Canadian Consortium on Neurodegeneration in Aging (CCNA), CAN-THUMBS UP Study Group

**Affiliations:** 1 Department of Neurosciences, University of California San Diego, 9500 Gilman Drive, MC 0949, 92037-0949 La Jolla, California USA; 2 Alzheimer’s Disease Cooperative Study, University of California San Diego, La Jolla, California USA; 3 Centre de recherche de l’Institut Universitaire de gériatrie du CIUSSS du Centre-Sud-de-l’Île-de-Montréal, Montreal, Quebec Canada; 4 Université de Montréal, Montreal, Quebec Canada; 5 Division of Neurology, University of British Columbia, Vancouver, British Columbia Canada; 6 Gait and Brain Laboratory, Lawson Health Research Institute, Parkwood Institute, London, Ontario Canada; 7 Schulich School of Medicine and Dentistry, Department of Medicine (Geriatrics), University of Western Ontario, London, Ontario Canada; 8 Rotman Research Institute and Baycrest Health Sciences, University of Toronto, Toronto, Ontario Canada; 9 Cognition & Technology Research Group, Institute on Aging and Lifelong Health, University of Victoria, Victoria, British Columbia Canada; 10 Sunnybrook Research Institute, Toronto, Ontario Canada; 11 Department of Health Research Methods, Evidence, and Impact, McMaster University, Hamilton, Ontario Canada; 12 Schulich School of Medicine and Dentistry, Department of Epidemiology and Biostatistics, University of Western Ontario, London, Ontario Canada

**Keywords:** Alzheimer’s disease, dementia, prevention, Brain Health PRO, NCT05347966

## Abstract

**Background/Objectives:**

CAN-THUMBS UP is designed as a comprehensive and innovative fully remote program to 1) develop an interactive and compelling online Brain Health Support Program intervention, with potential to positively influence dementia literacy, self-efficacy and lifestyle risk factors; 2) enroll and retain a community-dwelling Platform Trial Cohort of individuals at risk of dementia who will participate in the intervention; 3) support an open platform trial to test a variety of multidomain interventions that might further benefit individuals at risk of dementia. This manuscript presents the Brain Health Support Program Study protocol.

**Design/Setting:**

Twelve-month prospective multi-center longitudinal study to evaluate a fully remote web-based educational intervention. Participants will subsequently be part of a Platform Trial Cohort and may be eligible to participate in further dementia prevention clinical trials.

**Participants:**

Three hundred fifty older adults who are cognitively unimpaired or have mild cognitive impairment, with at least 1 well established dementia risk factor.

**Intervention:**

Participants engage in the Brain Health Support Program intervention for 45-weeks and complete pre/post intervention measures. This intervention is designed to convey best available evidence for dementia prevention, consists of 181 chapters within 8 modules that are progressively delivered, and is available online in English and French. The program has been developed as a collaborative effort by investigators with recognized expertise in the program’s content areas, along with input from older-adult citizen advisors.

**Measurements:**

This study utilizes adapted remote assessments with accessible technologies (e.g. videoconferencing, cognitive testing via computer and mobile phone, wearable devices to track physical activity and sleep, self-administered saliva sample collection). The primary outcome is change in dementia literacy, as measured by the Alzheimer’s Disease Knowledge Scale. Secondary outcomes include change in self-efficacy; engagement using the online program; user satisfaction ratings; and evaluation of usability and acceptance. Exploratory outcomes include changes in attitudes toward dementia, modifiable risk factors, performance on the Neuropsychological Test Battery, performance on self-administered online cognitive assessments, and levels of physical activity and sleep; success of the national recruitment plan; and the distribution of age adjusted polygenic hazard scores.

**Conclusions:**

This fully remote study provides an accessible approach to research with all study activities being completed in the participants’ home environment. This approach may reduce barriers to participation, provide an easier and less demanding participant experience, and reach a broader geography with recruitment from all regions of Canada. CAN-THUMBS UP represents a Canadian contribution to the global World-Wide FINGERS program (alz.org/wwfingers).

**Electronic Supplementary Material:**

Supplementary material is available in the online version of this article at 10.14283/jpad.2023.65.

## Introduction

Up to 40% of the population attributable risk of dementia resides in modifiable lifestyle factors (1), providing a strong rationale to develop personally tailored interventions to prevent dementia. There is a relatively untapped opportunity to delay or prevent dementia through the active management and lowering of modifiable risk factors, including vascular, diet, physical activity, sensory, cognitive and social engagement. This approach is increasingly recognized as the most tractable and immediate approach to potentially effective dementia prevention with the potential for an enormous public health impact (2). Even a modest delay of one year in the onset of dementia has been projected to result in almost 500,000 fewer new cases by 2050 (3) and could save the Canadian health care system $120 billion over the next 3 decades (4). Priorities for public health and lifestyle interventions have included those that promote brain resilience and address modifiable risk factors, an approach that can also be tailored to personalized risk profiles (5, 6).

### The role of education and engagement in dementia prevention

Prior studies have reported that even in the early stages of Alzheimer’s disease (AD), older adults can benefit from formal educational courses on dementia and that they can improve their knowledge of the disease, with beneficial effects on their mood and self-efficacy (7). Furthermore, web-based educational programs focusing on risks and protective factors have been found to improve dementia-related protection/risk profiles in middle-aged adults (8). Engaging in an educational program can potentially increase participants’ dementia literacy, empowerment, general self-efficacy, and engagement regarding ways to promote their brain health. Based on prior studies, this could have a positive risk reduction effect on its own while also improving participants’ readiness to change and increasing motivation to participate in further prevention of dementia studies.

### Personalized risk profiles and remote assessments

In considering the path to successful multidomain lifestyle interventions, key issues include effectively tailoring approaches to personalized risk profiles, addressing individual preferences, and attaining compliance with fidelity to the interventions to evoke behavioral change. It is increasingly possible to mobilize technology to more readily evaluate compliance and effects of lifestyle interventions, through the incorporation of wearable devices that track changes in activity levels and sleep patterns, and through app-based remote testing of cognitive function using paradigms of ‘burst’ cognitive testing (9, 10) and/or self-administered online cognitive testing. These measurement approaches can comprehensively inform how interventions are being adopted and also provide more sensitive measurements to detect whether there is an early and subtle slope of decline. Such early detection of decline could be used to design more powerful, personalized trials with the identification of those with the highest predicted risk and those with the lowest risk.

### Novel and better design of trials

Open platform trials (OPT), which include Master Protocols that have key shared design components, common inclusion and exclusion criteria, and common outcome measures, provide a novel and attractive approach to the conduct of randomized controlled trials (RCTs), with potential application to dementia prevention (11). Recruiting and engaging a pre-randomized Platform Trial Cohort (PTC) of individuals at increased risk of dementia through online activities shifts the recruitment paradigm away from its current model, which often results in operational challenges and trial slowdown (12). Furthermore, the pre-randomized data obtained from a PTC could serve to help validate the inclusion criteria, study design, and development of the Master Trial Protocol of the OPT.

### Study goal and aims

The Canadian Therapeutic Platform Trial for Multidomain Interventions to Prevent Dementia (CAN-THUMBS UP) is a comprehensive program that will 1) develop and evaluate an interactive educational program to positively influence dementia literacy and lifestyle risk factors; 2) enroll a PTC of individuals at risk of dementia who will participate in the educational intervention; 3) support an open platform with nested multidomain interventional clinical trials. Within CAN-THUMBS UP, the goal of the Brain Health Support Program (BHSP) Study is to develop and evaluate a 45-week web-based educational intervention, Brain Health PRO (BHPro), focused on dementia literacy, self-efficacy and modifiable lifestyle risk factors.

The primary aim is to:

- Evaluate within-person change in dementia literacy following participation in BHPro. Other study aims are to:

- Evaluate within-person change following participation in BHPro in: self-efficacy, attitudes toward dementia and its screening, individuals’ modifiable risk factors, cognition, as well as physical activity and sleep quality.
- Evaluate the online BHPro platform in relationship to: levels of engagement using the online program, the association between change in modifiable risk factors and change in cognition, and ratings of satisfaction, usability and acceptance.
- Develop a successful comprehensive national recruitment plan to fully enroll a PTC with engagement stakeholder groups including participants, citizen advisors, and community partners.

## Methods

### Study design

**Figure 1:**
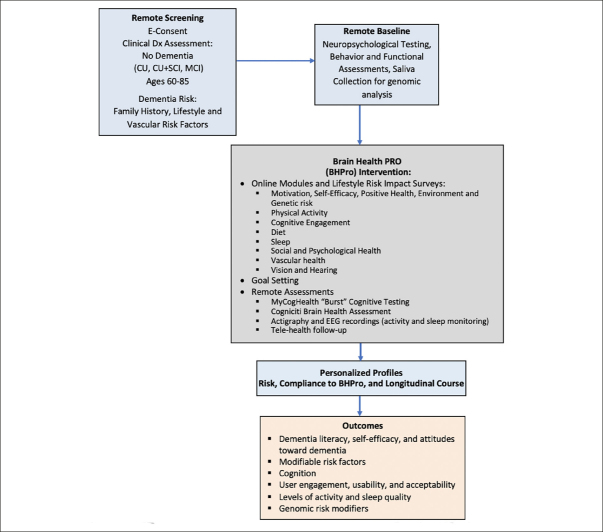
BHSP Study Schematic

The initial phase (Phase A) of CAN THUMBS-UP is a prospective 12-month multi-center longitudinal intervention study utilizing a one-group pretest-posttest design to evaluate BHPro. BHPro is a web-based formal educational program designed to increase dementia literacy, foster engagement, and convey best available evidence for lifestyle changes that can mitigate dementia risk. BHPro is available in both English and French and includes 181 chapters within 8 modules that are progressively delivered to participants each week. At the conclusion of the BHPro intervention, participants have the opportunity to provide a further consent to participate in nested multidomain clinical trials that are conducted using this PTC under separate protocols. The study schematic is shown in Figure 1.

Assessments occur at Screening, Baseline, Month 3, Month 6, Month 9, and Month 12. The schedule of events is shown in Table 1.

**Table 1:** Schedule of Events

**PROCEDURES**	**SCREENING (within 21 days of baseline)**	**BASELINE (+/− 2 weeks)**	**MONTH 3 (+/− 2 weeks)**	**MONTH 6 (+/− 2 weeks)**	**MONTH 9 (+/− 2 weeks)**	**MONTH 12 (+/− 2 weeks)**
TELE/VIDEO-CONFERENCE REMOTE PROCEDURES						
INFORMED CONSENT	X					
SOCIODEMOGRAPHICS	X					
MEDICAL/SURGICAL/FAMILY HISTORY	X			X		X
HEIGHT / WEIGHT / BMI	X					X
CLINICAL DEMENTIA RATING (CDR)	X					X
LAWTON BRODY INSTRUMENTAL ADLs	X					X
HEARING AND VISION ASSESSMENT	X					
CAIDE SCORE	X					X
REMOTE NEUROLOGICAL EXAM	X					X
FOLLOW-UP PHONE CALL (continued interest, satisfaction with BHPro, medical and diagnostic changes)			X	X	X	
*NEUROPSYCHOLOGICAL TEST BATTERY*						
MONTREAL COGNITIVE ASSESSMENT	X					X
LOGICAL MEMORY 1 and 2	X					
CRAFT STORY 21 Immediate and Delayed Recall AND DELAYED RECALL		X				X
ADAS-COG word recall, delayed recall, orientation		X				X
BRIEF VISUOSPATIAL MEMORY TEST-REVISED (BVMT-R)		X				X
ORAL SYMBOL DIGIT MODALITITES TEST		X				X
ORAL TRAIL MAKING TEST A AND B		X				X
DKEFS COLOR-WORD INTERFERENCE TEST		X				X
DKEFS VERBAL FLUENCY		X				X
BOSTON NAMING TEST (BNT), 15-item		X				X
MYCOGHEALTH BURST COGNITIVE TESTING		X	X	X	X	X
COGNICITI BRAIN HEALTH ASSESSMENT (BHA)		X		X		X
ONLINE FUNCTIONAL AND BEHAVIORAL ASSESSMENTS						
GERIATRIC DEPRESSION SCALE (GDS)		X				X
GENERALIZED ANXIETY DISORDER 7-ITEM SCALE (GAD-7)		X				X
MILD BEHAVIORAL IMPAIRMENT CHECKLIST (MBI-C)		X				X
SLEEP DISORDERS QUESTIONNAIRE		X				X
COVID-19 QUESTIONNAIRE		X				X
RESEARCH SATISFACTION SURVEY		X		X		X
OTHER REMOTE DATA COLLECTION AND MONITORING						
ACTIGRAPHY APPLICATION AND DATA COLLECTION		X				X
EEG WEARABLE INSTRUCTIONS AND DATA COLLECTION		X				X
SALIVA SAMPLE COLLECTION		X				
ONLINE BHPro ASSESSMENTS						
SELF-EFFICACY (The General Self-Efficacy scale, GSE)		X		X		X
DEMENTIA LITERACY (The Alzheimer’s Disease Knowledge Scale, ADKS)		X		X		X
ATTITUDE TOWARDS DEMENTIA AND DEMENTIA SCREENING (Parts B and D of the PRISM-PC Questionnaire)		X		X		X
USABILITY (web-based version System Usability Scale, SUS)				X		X
ACCEPTABILITY (Technology Acceptance Model questionnaire-adapted)				X		X
BHPro LIFESTYLE RISK QUESTIONNAIRES		X	X	X	X	X

### Participants and setting

Three hundred fifty older adults (ages 60–85) who are either cognitively unimpaired or have mild cognitive impairment (MCI) with at least 1 risk factor, and who meet inclusion and exclusion criteria are recruited from across all provinces and territories in Canada. Participants include English and French speaking individuals and geographical recruitment areas include both rural and urban. The research protocol is fully implemented remotely.

### Inclusion criteria

- Completion and documentation of the electronic Informed Consent Process (from the participant)
- Sufficient proficiency in English or French to undergo remote clinical and neuropsychological assessment and participate in an online educational program (i.e. a rating of 7 out of 10 on the Language Experience and Proficiency Questionnaire)
- Technical ability to participate in an online educational program and remote assessments (assessed by asking the participant the following questions: “Do you have access to a computer that connects to the internet?” and “Do you have the ability to send and receive emails?”)
- Sufficient vision and hearing to participate in an online educational program and to undergo remote clinical and neuropsychological testing (assessed by asking the participant a series of questions about their hearing/vision and investigator judgment)
- Ability to sit comfortably for a period of about 30 minutes
- Ages 60–85
- Meets criteria for No Dementia and meets criteria (according to Canadian Consortium on Neurodegeneration in Aging Criteria in Table 2) of one of the following: Cognitively Unimpaired (CU), Cognitively Unimpaired plus Subjective Cognitive Impairment (CU + SCI), Mild Cognitive Impairment (MCI)
- AND Classified as being at increased risk of dementia based on at least one of the following:
- First-degree family history of dementia
- Self-Reported or documented current and/or history at midlife (45–60 years) on any of the following lifestyle risk factors:
  - Hypertension (documented Systolic Blood Pressure > 140 mm Hg; OR physician diagnosis of hypertension; OR treatment for hypertension; OR other approaches to treatment (e.g., diet, exercise))
  - Hypercholesterolemia (documented total cholesterol > 6.5 mmol/L; OR physician diagnosis of hypercholesterolemia; OR treatment for hypercholesterolemia; OR other approaches to treatment (e.g. diet, exercise)
  - Body Mass Index > 30 kg/m2 (derived from NIH Metric BMI Calculator)
  - Physical Inactivity (active is defined as engaging in a minimum of 20–30 min of physical activity causing sweating and breathlessness, at least 2 times a week)
- Has a family physician or other healthcare provider and agrees to have the provider notified of participation in the study and incidental or other findings that may be clinically significant.

**Table 2:** Canadian Consortium on Neurodegeneration in Aging (CCNA) Clinical Diagnostic Criteria for Cognitively Unimpaired, Subjective Cognitive Impairment, and Mild Cognitive Impairment

**Group**	**Diagnostic Criteria**	**Operationalized as**
Cognitively Unimpaired (CU)	1. Normal age-, sex-, and education-adjusted performance on standardized cognitive tests	• Global CDR Score = 0
		• Logical Memory II Score=
		≥ 9 for 16 or more years of education
		≥ 5 for 8–15 years of education
		≥ 3 for 0–7 years of education
		• MoCA Total Score > 25
	2. No Subjective Memory Complaint	Answer “no” to one or both of the following questions: “Do you feel like your memory or thinking is becoming worse?” and “Does this concern you?”
	3. Does not meet clinical diagnostic criteria for Dementia	DSM-IV
Subjective Cognitive Impairment (SCI)	1. Normal age-, sex-, and education-adjusted performance on standardized cognitive tests	• Global CDR Score = 0
		• Logical Memory II Score=
		≥ 9 for 16 or more years of education
		≥ 5 for 8–15 years of education
		≥ 3 for 0–7 years of education
		• MoCA Total Score > 25
	2. Subjective Memory Complaint: Self-experienced persistent decline in cognitive capacity in comparison with a previously normal status and unrelated to an acute event	Answer “yes” to both of the following questions: “Do you feel like your memory or thinking is becoming worse?” and “Does this concern you?”
	3. Does not meet clinical diagnostic criteria for Dementia	DSM-IV
Mild Cognitive Impairment (MCI)	1. Concern regarding a change in cognition	Report from participant and/or informant
	2. Impairment in one or more cognitive domains	• Global CDR Score = 0.5
		• Logical Memory II Score =
		≤ 8 for 16 or more years of education
		≤ 4 for 8–15 years of education
		≤ 2 for 0–7 years of education
		• MoCA Total Score 13–24 inclusive
	3. Preservation of independence in functional abilities	• Lawton-Brody IADL Score >14/23
	4. Does not meet clinical diagnostic criteria for Dementia	• DSM-IV
		• Global CDR Score < 1.0

Note. Abbreviations: CDR, Clinical Dementia Rating; CCNA, Canadian Consortium on Neurodegeneration and Aging; DSM-IV, Diagnostic and Statistical Manual of Mental Disorders, Fourth Edition; MoCA, Montreal Cognitive Assessment; IADL, Instrumental Activities of Daily Living.

### Exclusion criteria

- Participants who, in the opinion of the investigator, are not able to complete trial procedures remotely or adhere to the schedule of study assessments will be excluded from study participation.
- Individuals whose understanding English or French is not sufficiently proficient for remote clinical assessment, neuropsychological testing and participation in an online educational program
- Participants who do not have sufficient vision and hearing for remote clinical assessment, neuropsychological testing, or participation in an online educational program
- Individuals who do not have the technical ability to participate in an online educational program. Technical ability is defined as having computer and internet access; ability to send and receive emails; ability to participate in remote assessments.
- Individuals who have a clinical diagnosis of Dementia
- Clinical Dementia Rating (CDR; telephone/video-conference administration) Score of >1
- Total Score on the Montreal Cognitive Assessment (MoCA; video-conference administration) <13

### Recruitment and screening

#### Recruitment procedures

Both centralized recruitment and site-specific activities are utilized. There are 7 regional sites across Canada, all with full time, dedicated study coordinators and regional Principal Investigators (PIs) to support participants. A nationwide recruitment campaign is implemented with the goal of bringing awareness of the study to more Canadians. The recruitment campaign utilizes specific strategies, such as geotargeted mailing and targeted social media advertising, to reach both men and women. Recruitment efforts using existing national and clinic registries and societies are also utilized. Potential participants learning about the study through the study’s advertising methods are invited to visit a dedicated study website [https://www.canthumbsup.ca], available in both English and French, where they can express their interest about the study and which initially establishes eligibility. Potential participants are assigned to a regional site coordinator who contacts them in follow-up.

#### Screening and consenting procedures

Consent is completed remotely through the electronic Informed Consent Process (e-Consent) in accordance with local research ethics board standards, before any study-related activities are conducted. A study coordinator schedules a phone or video call with the participant to explain the details of this study and provide the participant the opportunity to ask questions before sending them the e-mail link to complete the e-Consent. The e-Consent provides an interactive and engaging informed consent experience with built in quiz questions which must be answered correctly in order to demonstrate comprehension. A hard copy of the informed consent form may be mailed/e-mailed to the participant by site staff according to site policy or participant preference.

The screening visit is conducted remotely via secure video-conference technology. Screening assessments, listed in Table 1, are conducted via video call with the participant and study partner. For assessments that utilize visual stimuli, the stimuli are displayed on the participant’s screen using the Screen Share function on the videoconference platform. Participants are asked about current vascular risk factors and history at mid-life to capture both a current and mid-life Cardiovascular Risk Factors, Ageing and Dementia (CAIDE) risk score. Following completion of the screening assessments, the regional site PI is responsible for confirming participant diagnosis and eligibility for enrollment in the study.

#### Study partner (optional)

The role of the study partner (optional) is to answer questions about the participant and his or her health, memory, daily functioning, and behavior. The study partner is also asked to provide informed consent for their role. Not having a study partner available does not exclude the participant from joining this study. If no study partner is available, the global CDR score is determined by rater and site PI consensus using all other available information and best clinical judgement.

### Early withdrawals

Participants are free to withdraw from study participation at any time, for any reason, and without prejudice.

Study discontinuation for an individual participant may occur in the following circumstances: 1) withdrawal of informed consent by the participant, 2) adverse event or other significant medical condition which, in the opinion of the Investigator, renders it necessary to remove the individual from study participation, or 3) any other occurrence that, in the Investigator’s opinion, makes continued participation contrary to the participant’s best interests.

Participants who have logged in and completed the registration process for BHPro but choose to withdraw and/or discontinue participation in the BHPro intervention for any reason are offered the opportunity to continue on the protocol with further visits per protocol up to the end of the study with their ongoing consent. If a participant declines to complete further study visits per protocol, an End of Study Remote Visit is completed as close as possible to the time of study discontinuation.

### Retention

Strategies implemented to promote retention of participants include 1) conducting follow-up telephone calls with participants every 3 months, 2) having regional study coordinators available to answer participant questions via email or telephone, and 3) providing access to a help desk for technical assistance with the online intervention.

### Intervention

#### Brain Health PRO (BHPro)

The BHPro Intervention is a 45-week, multidomain, web-based formal educational program designed to increase dementia literacy, foster engagement, and convey best available evidence for lifestyle changes that can mitigate dementia risk. Participants receive instructions on how to register and participate in the program at the Baseline study visit.

#### Program content

Participants register for the program and obtain a personal password. Registration includes demographic questions and lifestyle questionnaires including general questions related to socio-demographic profile, health, and lifestyle.

BHPro is organized into the following 8 content modules: 1) general information on cognition, dementia, genetic risk and healthy lifestyle, 2) physical activity, 3) cognitive engagement, 4) diet, 5) sleep, 6) social and psychological health, 7) vascular health, 8) vision and hearing.

The program content is provided progressively to deliver new weekly content (i.e., module chapters) of approximately 40 minutes. Participants have access to new content on 4 modules each week (10 minutes/module) through an email push notification, as an approach to maintain interest. Participants are invited to go over the material at their own rate over the week.

Information is organized as chapters within the modules, which contain scientific evidence, explanation of principles and mechanisms that underlie positive effects, specific recommendations (e.g., type and dose of prescribed physical exercises), suggestions to improve behavior in everyday life and typical barriers and tips to circumvent them. Content format includes visual and auditory text, pictures, animations, quizzes, questionnaires and interactive exercises.

#### Personalized profiles on modifiable lifestyle risk factors

Participants are asked to complete brief online lifestyle risk questionnaires approximately every 3 months. Questions are related to the content in the program modules (i.e., sleep, diet, physical activity, cognitive engagement, social and psychological health, vascular health, vision and hearing). A personalized profile is then developed for each participant based on their responses to the lifestyle questionnaires and is provided back in a personalized profile display. Participants are also asked to set prioritize topics and set goals based on their risk profile and to re-visit their priorities and goals approximately every 3 months. Participants are encouraged to focus on the content modules that are related to their risk profile. Although participants have access to all content modules within the program, participants receive more chapters for topics that are prioritized. After 45-weeks of content are progressively delivered, participants have access to all content until their end of study visit at Month 12.

### Assessment outcomes

#### Primary outcomes

The primary outcome is change in dementia literacy following participation in the study, as measured by the Alzheimer’s Disease Knowledge Scale (ADKS). The ADKS is a 30-item, true/false scale designed to assess knowledge about AD among laypeople, patients, caregivers, and professionals (13). It covers risk factors, assessment and diagnosis, symptoms, course, life impact, caregiving, and treatment management.

#### Secondary outcomes

Secondary outcomes include change following participation in the study in self-efficacy, as measured by the General Self-Efficacy Scale (GSE) (14), engagement using the online program, user satisfaction ratings, and evaluation of usability (System Usability Scale) (15) and acceptance (adapted Technology Acceptance Model Questionnaire) (16). Table 1 provides a complete list of outcome measures and assessment timepoints.

#### Exploratory outcomes

Exploratory outcomes include 1) change in attitudes toward dementia and dementia screening, as measured by Sections B and D of the Perceptions Regarding Investigational Screening for Memory in Primary Care (PRISM-PC) (17), 2) change in modifiable risk factors, as measured by BHPro Lifestyle Risk Questionnaires, and 3) change in global cognition, memory, processing speed, and executive functioning composite scores derived from the Neuropsychological Test Battery. We also evaluate change in performance on self-administered cognitive assessments using the web-based Cogniciti Brain Health Assessment (18) and the MyCogHealth mobile application (9, 10). The Cogniciti Brain Health Assessment (BHA) is a self-administered online cognitive screening tool developed for use with middle-aged and older adults. Comprised of 4 cognitive tests (Spatial Working Memory, Stroop Interference, Face-Name Association, and Number-Letter Alternation), the BHA can be completed in approximately 20 minutes and has demonstrated adequate reliability and construct validity (18), and criterion validity for detecting MCI in older adults (19). Participants complete the BHA unsupervised on their personal computers at Baseline, Month 6, and Month 12. The MyCogHealth mobile application is included to provide very brief intensive measurement “bursts”, which capture individual difference in cognitive performance variability and learning effects. Participants complete 5 assessment bursts (each comprising 2 testing sessions daily across 7 consecutive days), spaced approximately 3 months apart, over 12 months. Each testing session takes approximately 5 minutes to complete and includes 3 cognitive tests (Symbol Match, Dot Memory, Trail-Making A and B) and several brief questions assessing state factors at the time of testing (e.g., mood, fatigue, subjective cognitive ability). This measurement approach has demonstrated good feasibility, reliability, and construct validity with older adults (10).

Changes in levels of physical activity and sleep quality are measured by actigraphy and electroencephalography (EEG) wearable devices. Overnight EEG recording is assessed for 3 consecutive nights at Baseline and Month 12 using the MUSE S Headband (Interaxon Inc, Toronto, Ontario, Canada), a commercially-available consumer EEG headband with 5 dry electrodes, 2 on the forehead, 2 above the ears, plus 1 reference electrode. Physical activity and sleep monitoring using actigraphy (AX3, Axivity, Newcastle, United Kingdom) are also assessed at Baseline and Month 12. Participants are instructed to wear the Axivity AX3 on their non-dominant hand continuously for 14 days, even when bathing or swimming. During the 14 days, participants are asked to track their work and sleep hours in a diary.

National recruitment success is measured by enrollment rates, screen fail rate and reasons, and projected versus actual enrollment rates. Saliva is collected for de-identified DNA testing to characterize the distribution of age adjusted Polygenic Hazard Scores (PHS) within the cohort. At Baseline, a saliva sample collection kit (OG-500 kit, DNA Genotek, Kanata, Ontario) is sent to each participant for extraction of DNA. A PHS will be derived from a planned panel of single nucleotide polymorphisms that describes age of onset and AD risk (20).

### Sample size

With a baseline (t1) sample size of 350 participants and an anticipated 20–30% range of drop-out rates, there will be between 245 (with 30% dropout) and 280 (with 20% dropout) completed participants at t2 (end of intervention by week 52). All calculations are for 80% power and 5% two-tailed alpha. We considered small (d=0.2) and medium (d=0.5) Effect Sizes for 4 different t1–t2 correlations (rt1-t2) in the primary outcome variable: r=0.2, 0.3, 0.4 and 0.5. A final sample of n=280 will allow us to detect an ES ≥0.2 if rt1-t2≥0.3, while a final sample of n=245 will allow us to detect an ES ≥0.2 if rt1-t2≥0.4. We are aiming to recruit 50% women to allow for later stratification of analyses to assess for sex differences.

### Statistical analysis

In our one-group pretest-posttest design the major interest is within-person change in the primary and secondary outcomes over time (response variables) in the presence of one or more explanatory variables that are categorical or continuous. The data will be analyzed using a mixed model for repeated measures approach (MMRM). This approach will treat sites and participants as random effects, while observational time and their characteristics such as sex and age as fixed effects. Potential effect modifications will be examined using this MMRM approach with variable selection based on Akaike/Bayesian information criterion. The MMRM approach will effectively handle missing data, with the assumption that data are missing at random. In this approach, information on missing observations is recovered from the observed outcomes via the within-patient correlation structure. In contrast to the flawed method of last observation carried forward (21), which uses only one data point, a MMRM analysis uses all the available data to compensate for the data missing on a particular participant.

The primary analysis population will be an intention to treat (i.e., participants with at least one outcome measure will be included in the analysis). Sex-stratification will be used in analyses as appropriate. Where appropriate and available, sex-specific normative data will be used in the scoring of cognitive tests. In assessing the psychometric properties of composites, the cohort will be divided into groups based on sex.

### Data management and monitoring

De-identified study data is stored on the Longitudinal Online Research and Imaging System (LORIS), a secure server at the McGill Centre for Integrative Neuroscience, McGill University, Montreal, Quebec. A data monitoring committee is not instituted for this study. Internal processes implemented to promote data quality include training of site personnel, direct data entry into electronic case report forms, and data validation edit checks within LORIS. Additionally, a supervising neuropsychologist is undertaking a review of audio recordings of 20% of Screening test administrations (MoCA, Logical Memory) and 10% of Baseline and Month 12 neuropsychological test battery administrations to ensure accuracy of administration, scoring, and data entry. Inconsistent and questionable data detected during the data entry or validation process will be queried and resolved prior to the data lock and analysis.

### Adverse events

Risks associated with participation in the BHSP study are minimal. Participation does not include any invasive procedures. For the purposes of reporting on this study, only adverse events (AEs) associated with study procedures are tracked. AEs are assessed by the site PI and documented in LORIS.

### Access to data

CAN-THUMBS UP and the BHSP are a program within the broad-based Canadian Consortium on Neurodegeneration in Aging (CCNA) (22). In order to access the data generated in this study, researchers must agree to abide by the CCNA Publication and Data Access Committee (PDAC) policy, a document prepared by the CCNA PDAC, available for download at www.ccna-ccnv.ca. Access to and analyses of study data stored in LORIS may be granted to qualified persons 12 months after the principal paper answering primary research questions are published. Such requests will be made via email to CCNA [ccna.admin@ladydavis.ca] or via the LORIS Data Access Module.

During the screening visit, demographic questions include a question with Indigenous identity response options (First Nations, Inuit and/or Metis). Data access requests that propose to utilize this or other Indigenous identifiers will undergo additional review to ensure that meaningful and respectful engagement with relevant Indigenous Peoples is integrated throughout the research process.

### Platform Trial Cohort

All participants who meet BHSP Study eligibility criteria will be enrolled in the PTC. Once participation in the BHSP study has ended, participants may be offered potential participation in future multidomain intervention RCTs of the CAN-THUMBS UP program under separate protocols. For participants who are interested and meet inclusion criteria for specific RCTs within the OPT, a separate informed consent will be signed prior to participation.

### Participant and public involvement

BHPro was developed as a collaborative effort by CCNA investigators with recognized expertise in the program’s content areas, along with input from older-adult citizen advisors. CAN-THUMBS UP established a citizen advisor group (CAG), comprised of 9 older adults (6 women, 3 men) from the community in 3 provinces, with the goal of supporting co-creation efforts. CAG members met regularly for over a year during the BHPro development phase to review content and provide feedback to content experts in the development process, in order to best meet the needs of CAN-THUMBS UP’s target audience. In addition, BHPro was refined and updated based on feedback from focus-groups that were conducted as part of a 3-month pilot study designed to assess usability and accessibility.

### Ethics and dissemination

#### Research ethics approvals

This study is conducted in compliance with International Conference on Harmonisation of Good Clinical Practice and all applicable regulatory requirements. CAN-THUMBS UP: BHSP has undergone review and approval from the research ethics committees/boards of the University of British Columbia (#H22-00563); University of Victoria (#H20-03475); Centre intégré universitaire de santé et de services sociaux du Centre-Sud-de-l’Île-de-Montréal (CER VN 21-22-16); Horizon Health Network (#2022-3096); Vitalité Health Network; Cape Breton University Centre of Excellence for Healthy Aging (#2022-035). All sites located within Ontario received centralized provincial approval from Clinical Trials Ontario (#3964) and also from their local research ethics committees/boards: Baycrest, Parkwood Research Institute, and Sunnybrook Research Institute. Protocol amendments will be tracked, dated, reviewed, and signed off by all Co-PIs. Site PIs will obtain approval from their local REBs for all protocol amendments.

#### Dissemination plan and authorship

Results of this study will be published in peer-reviewed journals, and presented to local stakeholders, and at provincial, national, and international conferences. In accordance with the International Committee of Medical Journal Editors’ standards, authorship of publications resulting from this study will be based and accurately reflect the academic contribution of individuals to the design and implementation of the trial, analysis of the data and preparation of the manuscript. No researcher shall include identifiable personal health information in any publication or presentation. CAN-THUMBS UP represents a Canadian contribution to the global WorldWide FINGERS program (23).

## Discussion

This paper presents the protocol for the BHSP Study, a 12-month prospective multi-center longitudinal intervention study to evaluate a fully remote web-based educational program, BHPro. Through BHPro, individuals engage in an innovative, accessible, and multimodal platform conveying evidence-based information and guidance on lifestyle risk factors including, vascular, dietary, sleep, physical activity, cognition and social engagement. Following participation in this study, participants will be part of a Platform Trial Cohort and may be eligible to participate in further dementia prevention clinical trials.

The BHSP Study includes individuals age 60–85 who are cognitively unimpaired or have MCI, and have at least 1 well established dementia risk factor. Importantly, older adults who participate in an educational intervention could potentially increase their dementia literacy, empowerment, general self-efficacy and engagement regarding ways to promote their brain health. Targeting individuals 60 years and older is in alignment with the World-Wide FINGERS collaboration and several of its large scale dementia prevention trials such as FINGER (24) and U.S. POINTER (25). Including participants with MCI, which is an important risk factor for dementia, will allow BHPro to be evaluated across a broader risk continuum.

This fully remote online research protocol provides an accessible approach to research, with all study activities being completed in the participants’ home environment. Previous studies have demonstrated that in-home assessments are feasible and can be implemented with acceptable study dropout rates (26). In the BHSP Study, data is collected by utilizing adapted remote assessments with accessible technology (e.g., videoconferencing, electronic informed consent, cognitive testing via computer and mobile phone, wearable devices to track physical activity and sleep, self-administered saliva sample collection). Employing technology for remote data collection provides a timely opportunity to engage with participants and to advance research in dementia prevention in times of limited/restricted in-person engagement including the global coronavirus (COVID-19) pandemic.

The BHSP Study includes a comprehensive nationwide recruitment strategy designed for a virtual environment. Methods such as geotargeted mailing, targeted social media advertising, and a central study website are implemented with the goal of bringing awareness of the study to more Canadians. Seven regional study sites across Canada, each with a full time, dedicated study coordinator and regional PI are available to enroll and support participants.

There are foreseeable limitations of this study. First, participants are required to have the technical ability to participate (e.g., access to technology and the ability to use the technology). To address this, a help desk is provided for technical assistance with BHPro, study staff are available to answer questions via phone or email, and study phones are provided to participants without a smartphone. Importantly, early data from initial pilot testing indicates the BHPro platform meets the needs and abilities of older adults in a virtual setting. Pilot study focus groups and user experience questionnaires indicate excellent usability of BHPro, with all dimensions rated in the positive range.

A second limitation, is this study utilizes a one-group pretest-posttest design, in which the major interest is within-person change over time. As such, it lacks a concurrent randomized non-intervention comparator arm. Consequently, changes in the primary outcome will need to be interpreted in the context of background knowledge of expected changes in natural history that are not directly observed in this study.

The fully remote approach used in this study has the potential to reduce barriers to participation, provide an easier and less demanding participant experience, and reach a broader geography with recruitment from all regions of Canada. Once validated, the BHPro intervention may potentially serve as a feasible and ethical control condition for multiple other prevention interventions around the world. This study is currently ongoing at the time of submitting this manuscript (February 2023). Recruitment started in April 2022 and enrollment was completed in December 2022. The study is expected to be completed in January 2024.

## Electronic Supplementary Material

Supplementary material, approximately 185 KB.

Supplementary material, approximately 13.3 KB.

## References

[1] Livingston G (2020). Dementia prevention, intervention, and care: 2020 report of the Lancet Commission. Lancet.

[2] Livingston G (2017). Dementia prevention, intervention, and care. Lancet.

[3] Navigating the Path Forward for Dementia in Canada: The Landmark Study Report #1. 2022, Alzheimer Society of Canada.

[4] Ostbye T, Crosse E (1994). Net economic costs of dementia in Canada. CMAJ.

[5] Organization, W.H., Risk Reduction of Cognitive Decline and Dementia: WHO Guidelines. 2019, WHO Guidelines Approved by the Guidelines Review Committee: Geneva. 31219687

[6] Stern, Y., et al., Whitepaper: Defining and investigating cognitive reserve, brain reserve, and brain maintenance. Alzheimer’s &amp; Dementia, 2018. 10.1016/j.jalz.2018.07.219 10.1016/j.jalz.2018.07.219 PMC6417987 30222945

[7] Fitzsimmons S, Buettner LL (2003). Health promotion for the mind, body, and spirit: a college course for older adults with dementia. Am J Alzheimers Dis Other Demen.

[8] Anstey KJ (2015). Body brain life: A randomized controlled trial of an online dementia risk reduction intervention in middle-aged adults at risk of Alzheimer’s disease. Alzheimers Dement (N Y).

[9] Brewster P (2020). Intensive measurement of cognition to support early detection of cognitive change in individuals at risk of dementia. Alzheimer’s &amp; Dementia.

[10] Brewster PWH (2021). Feasibility and Psychometric Integrity of Mobile Phone-Based Intensive Measurement of Cognition in Older Adults. Exp Aging Res.

[11] Woodcock J, LaVange LM (2017). Master Protocols to Study Multiple Therapies, Multiple Diseases, or Both. N Engl J Med.

[12] Fargo KN (2016). The crisis in recruitment for clinical trials in Alzheimer’s and dementia: An action plan for solutions. Alzheimers Dement.

[13] Carpenter BD (2009). The Alzheimer’s Disease Knowledge Scale: development and psychometric properties. Gerontologist.

[14] Schwarzer R, Jerusalem M (2010). The general self-efficacy scale (GSE). Anxiety, stress &amp; coping.

[15] Brooke J (1996). SUS-A quick and dirty usability scale. Usability evaluation in industry.

[16] Venkatesh V, Davis FD (2000). A theoretical extension of the Technology Acceptance Model: Four longitudinal field studies. Management Science.

[17] Boustani M (2008). Measuring primary care patients’ attitudes about dementia screening. Int J Geriatr Psychiatry.

[18] Troyer, A.K., et al., Development and evaluation of a self-administered on-line test of memory and attention for middle-aged and older adults. Frontiers in Aging and Neuroscience, 2014. 6. 10.3389/fnagi.2014.00335 10.3389/fnagi.2014.00335 PMC4261807 25540620

[19] Paterson TSE (2021). Accuracy of a Self-Administered Online Cognitive Assessment in Detecting Amnestic Mild Cognitive Impairment. The Journals of Gerontology: Series B.

[20] Desikan RS (2017). Genetic assessment of age-associated Alzheimer disease risk: Development and validation of a polygenic hazard score. PLoS Med.

[21] Molnar FJ, Hutton B, Fergusson D (2008). Does analysis using “last observation carried forward” introduce bias in dementia research?. CMAJ.

[22] Chertkow, H., et al., The Comprehensive Assessment of Neurodegeneration and Dementia: Canadian Cohort Study. Can J Neurol Sci, 2019: p. 1–13. 10.1017/cjn.2019.27 10.1017/cjn.2019.27 31309917

[23] Kivipelto M (2020). World-Wide FINGERS Network: A global approach to risk reduction and prevention of dementia. Alzheimers Dement.

[24] Kivipelto M (2013). The Finnish Geriatric Intervention Study to Prevent Cognitive Impairment and Disability (FINGER): study design and progress. Alzheimers Dement.

[25] Baker LD (2020). U.S. POINTER (USA). Alzheimer’s &amp; Dementia.

[26] Sano M (2019). A randomized clinical trial to evaluate home-based assessment of people over 75 years old. Alzheimers Dement.

