# Supplementary material for: Protocol for the Brain Health Support Program Study of the Canadian Therapeutic Platform Trial for Multidomain Interventions to Prevent Dementia (CAN-THUMBS UP): A Prospective 12-Month Intervention Study
Source: J Prev Alzheimers Dis. 2023 Jun 15:1–11. Online ahead of print. doi: 10.14283/jpad.2023.65 (PMC10258470; doi:10.14283/jpad.2023.65)
Supplement: Supplementary file 1 — Supplementary material, approximately 185 KB. [file 42414_2023_227_MOESM1_ESM.docx]

**
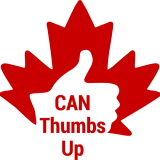
**

**The CTU: BHSP Study**

**Participant Information and Consent Form**

**Study Title:** The Canadian Therapeutic Platform Trial for Multidomain Interventions to Prevent Dementia (CAN-THUMBS UP or CTU): Brain Health Support Program Intervention (BHSP)

**Study Conducted by:** Canadian Consortium on Neurodegeneration and Aging (CCNA)

**Study Site:** XXXXXX

**Principal Investigator:** XXXXXX

**Study Coordinator:** XXXXXX

##

**INVITATION**

You are being invited to participate in a research study to help us assess how well a Brain Health Support Program (BHSP) allows us to engage with participants, increase their knowledge about dementia, and improve lifestyle risk factors in those at risk of dementia. This consent form describes a research study and your role as a research participant. This document is meant to inform you about the possible risks and benefits of the research study. It tells you about other options that may be available to you and your rights as a research participant. Please read this consent form carefully. Do not hesitate to ask the study investigator or study team any questions you may have about the study or the information given below.

**YOUR PARTICIPATION IS VOLUNTARY**

Your participation in this study is entirely voluntary. Please take as much time as you need to discuss the study with your doctors, family, and friends. The decision to participate or not is yours to make. If you choose to participate, you have the right to withdraw from the study at any time.

**WHO IS CONDUCTING THIS STUDY?**

The CAN-THUMBS UP (CTU) study is being conducted by the Canadian Consortium for Neurodegeneration in Aging (CCNA), through a grant from the Canadian Institutes of Health Research (CIHR). It is funded by multiple partners (CIHR, Alzheimer’s Society of Canada, Centre for Aging and Brain Health Innovation, Public Health Agency of Canada, Fonds de recherche du Québec – Société et culture).

**WHY IS THIS STUDY BEING DONE?**

**Background**

Prior studies have shown that programs that focus on promoting brain health and managing lifestyle risks (such as poor diet, obesity, physical inactivity, sleep issues, loneliness) may help in preventing or lowering the risk of dementia. To address this, we have developed the CAN-THUMBS UP program to conduct studies that target lifestyle risk and focus on dementia prevention. This program will also help us to recruit a large group of older-adults (called a study group) who are interested in participating in these types of studies. During the initial phase of CAN-THUMBS UP, we will evaluate how well a 45-week online Brain Health Support Program (BHSP), called Brain Health PRO (BHPro), allows us to engage with participants, increase their knowledge about dementia, and improve lifestyle risk factors. As a participant in this study, we will collect data and evaluate you as you complete the 45-week online BHPro. Once the BHPro program has ended, you will remain in the CAN-THUMBS UP study group and may be eligible to participate in future studies on lifestyle risk and dementia prevention. We will use the information that was collected during this study to help us design future studies that have more intensive lifestyle interventions (e.g. exercise training, diet, brain training). If we determine that you may be eligible for a future study, we may re-contact you at a later time to see if you are interested in participating in another study.

**What Is the Purpose of The Study?**

This study will help us evaluate how well BHPro is working and help us recruit a large group of older-adults who are interested in participating in studies that focus on promoting brain health and managing lifestyle risks that may help in preventing or lowering risk of dementia.

**Who Can Participate in This Study?**

You may be able to participate in this study if:

- You are 60-85 years of age
- You have either no memory loss or mild memory loss
- You are at increased risk of dementia due to:
- Having a first-degree family history of dementia, OR
- Having a current or past history of any of the following: hypertension (high blood pressure), hypercholesterolemia (high cholesterol), high body mass index (BMI), or physically inactive lifestyle
- You have the technical ability to participate in the online program and remote assessments (i.e. computer with internet access; ability to send and receive emails; ability to complete remote assessments)
- You have a family doctor or other healthcare provider and agree to have your provider notified of your participation in this study

**Who Should Not Participate in This Study?**

You will not be able to participate in this study if:

- You have a clinical diagnosis of dementia
- Your English or French is not sufficient to undergo clinical and neuropsychological assessments (tests of memory and thinking) and to participate in an online educational program
- You have vision or hearing impairments that would prevent you from participating in an online educational program

The study staff will discuss with you the full list of inclusion and exclusion criteria before determining whether you are eligible to participate in this study.

**WHAT IS INVOLVED IN THIS STUDY?**

Your participation in this study will last about 12 months and all activities will be done at home and online. You are being asked to take part in this study because you are an adult between **60 and 85** years old with either **no memory loss** or **mild memory loss**. Up to 350 adults from across Canada will take part in this study. There are up to 7 regional research sites located in British Columbia, Ontario, Quebec, Nova Scotia, and New Brunswick.

You will need regular access to a computer with internet access to participate in the online Brain Health Support Program, called Brain Health PRO (BHPro). Some tests will also be completed on a smartphone. If you do not have your own smartphone, the study team may be able to provide you with a phone that you can use during your participation in this study. Throughout your participation in the study, research staff will be available by phone or video call. They will answer any questions that you might have or to help with any technical difficulties. The video platform that will be used in this study to conduct visits is Zoom. Zoom visits will be conducted under [Insert Site Name] Zoom license, which meets provincial privacy standards. Please view the privacy policy for Zoom here: <https://zoom.us/privacy>.

Over the next 12 months, your participation in this study will involve:

**Study visits by phone/video-call** with a study team member:

- Give information about yourself and your medical history
- Take tests of memory and thinking
- Answer questions about your memory, mood, and daily activities
- Receive a brief neurological exam

Participate in **Brain Health PRO,** for 45 weeks:

- Register online and answer questions about yourself and daily lifestyle
- Spend about 40 minutes a week participating in the program
- Answer questions about how well the program is working
- Answer questions about your daily lifestyle and set goals for yourself

You will also be asked to complete the following activities **at home**:

- Take brief online tests of memory and thinking on a smartphone and computer
- Wear a wristwatch device (called an actigraph) to track your physical activity and sleep for 28 total days (14 days at the beginning and 14 days at the end of the study)
- Wear a sensor headband that tracks your sleeping for 6 total nights (3 nights at the beginning and 3 nights at the end of the study)
- Collect a saliva (spit) sample with a kit mailed to your home

If you have someone close to you (spouse, friend, relative), with whom you have contact on a weekly basis, they may also be asked to participate in this study as a “Study Partner” and will:

- Answer questions about your memory and everyday functioning

If you do not have a Study Partner, you will still be able to participate in this study.

**Study Visits by Phone/Video-Call**

A study team member will contact you by phone and/or video conference to complete your study visits from home. All study devices and materials will be mailed to your home ahead of your scheduled phone/video-call. We will provide you with pre-paid envelopes to mail everything back to the study site.

**If You Decide to Join This Study: Specific Procedures:**

**Visit 1 (Screening)**

During your **Visit 1** video call, we will determine your eligibility to enroll in this study. You must first review and complete the electronic consent process before any other screening procedures can be completed. The study staff will explain all of these procedures and answer any questions that you and your study partner (if you have one) may have.

The total approximate time to complete the Screening procedures is 3 - 3.5 hours. These may be completed in one video call or it may be done over a couple of days. The following tests and procedures will be performed during this screening video call with a study team member:

- **Demographic Questions:** You will be asked to give personal information about yourself, such as your name, birth month and year, etc.
- **Medical History:** Your general medical history will be recorded. We may request additional records if we have questions about your medical history. We will ask you to sign a ‘Release of Information’ form to obtain these medical records, if needed.
- **Medications:** We will review your current medications.
- **Height and Weight:** We will ask you to tell us your approximate height and weight.
- **Memory Testing:** You will complete brief tests on your memory and thinking. Most of these tests will involve question and answer format but some may involve paper and pencil. You will be able to skip any questions that you are uncomfortable answering.
- Your study partner (if you have one) will also be asked questions about your memory and thinking skills.
- **Neurological Exam**: A study doctor will evaluate you and complete a brief neurological exam over video-call. The study doctor or research staff will let you know what will happen during the exam.
- We will evaluate your eligibility to participate in this study.

**Visit 2 (Baseline)**

If following the screening you are determined to be eligible to participate in this study, you will be asked to complete the following Baseline study activities over video-call with a study team member. These study activities may be completed over multiple days.

The total approximate time to complete the Visit 2 study activities is 2.5 hours. The baseline study activities include:

- **Medical History:** We will review any changes in health that you may have experienced since the last study call.
- **Memory Testing:** You will complete tests of memory and thinking. Most of the tests will be in question and answer format, but some will involve paper and pencil and some will be done on computer. All tests that have to be completed on paper will be mailed to you.
- **Questionnaires:** You will also be asked questions about your memory, daily activities, mood, sleep behavior, and the impact of COVID-19 on you. Your study partner (if you have one) will be asked questions about your memory, mood and daily functioning.

During your video-call, a study team member will also give you detailed instructions for completing the following **study activities at home**:

- **Saliva Sample**: We will send you a kit to collect a saliva (spit) sample to study your genes. The sample will be stored for analysis of the genetic content known as DNA, or deoxyribonucleic acid, in order to learn about genetic information that may increase a person’s risk for developing dementia. It is for research purposes and is not a diagnostic test; therefore, you will not receive results of this testing.
- **Smartphone Memory Testing**: A study team member will show you how to use your smartphone for testing during your Visit 2 video-call. If you are using your own smartphone, a team member will help you download and install an application called MyCogHealth. If you are using a study provided smartphone, the smartphone will already have MyCogHealth installed. You will use the MyCogHealth application to complete short (5-10 minutes) tests of your memory and thinking and brief survey of your mood twice a day (morning and evening). You will complete your first testing session during your video-call visit and then will complete testing on your own for 6 days in a row. You will receive a notice on your smartphone when it is time to complete these tests. Study Staff will also be available by phone or video-call to answer any questions that you might have or if you experience any technical difficulty. You will be able to skip any questions that you are uncomfortable answering.
- **Computer Memory Testing:** During your Visit 2 video-call, a study team member will give you detailed instructions for completing brief tests (approx.20 minutes total) of your memory and thinking (called the Cogniciti Brain Health Assessment) online on your computer. You will be able to skip any questions that you are uncomfortable answering.
- **Physical Activity Monitoring:** You will apply a wristwatch-like device (called an actigraph) to your wrist and you will be asked to wear it 24 hours a day (except while bathing or swimming) for 14 days. It is not a tracking device, nor does it record heart rate – it simply measures the amount you are moving at any given time. At the end of 14 days you will remove the device and mail it back to the study site using a pre-addressed, stamped envelope that will be provided to you. You will also be asked to keep a sleep and work diary for these 14 days.
- **Sleep Recording:** You will also receive instructions during your Visit 2 video-call on how to wear an EEG (electroencephalogram) sleep headband and use the study provided iPod to record your sleep for 3 nights in a row. The headband is a commercially-available device that has sensors (on the forehead and behind the ears) to detect and measure sleep signals. After 3 nights of recording, you will mail the headband and iPod back to the study using a pre-addressed, stamped envelope that will be provided to you by the study. You will also be asked to fill out a survey to collect your feedback about the sleep headband after wearing it for the 3 nights.

At Home Sleep and Activity Testing devices shown in Figures 1-2.

**
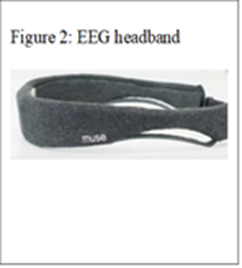
**
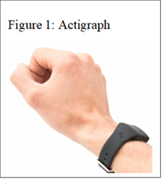


**Brain Health Pro (BHPro)**

You will be given detailed instructions on how to register and participate in the online BHPro intervention on a computer during your Visit 2 video-call with a study team member. You will then receive an e-mail to log-in and complete the registration for BHPro within 1 week after completing your Visit 2 (baseline) study activities. You will need regular access to a computer with internet and a personal e-mail address in order to participate in the program.

You will need a valid e-mail address and password to login and participate in the program. You will also be asked to enter your preferred name (this can be a nickname), which will appear on your personal profile on the website. Your chosen name and e-mail address will only be used for your participation in the program and will not be included in the data we collect for the research study.

BHPro is an educational program designed to teach you about dementia. It aims to provide best available evidence for lifestyle changes that can help lower dementia risk, and to provide you with specific recommendations for positive lifestyle changes (e.g., physical exercises to try at home, healthy recipes). The program was developed as a collaborative effort by Canadian investigators who have expertise in the different areas of dementia prevention, along with input from older-adult citizen advisors. As a part of this study you will be asked to complete 45 weeks of the program.

**BHPro Program Content**

The first step is to register for the program. Registration will include answering questions about yourself (such as your birth month and year, language, email address) and choosing a nickname and password. Next you will be presented with a brief video explaining the program, followed by questions about your lifestyle, health, daily activities and mood. Registration will take about 45 minutes to 1 hour to complete.

BHPro is organized into 8 different content areas (called “modules”):

1. Introduction (the first module is an introductory module that will provide an outline of the program and provide information on motivation, positive health, dementia risk and aging)
2. Physical activity
3. Cognitively (brain) stimulating activities
4. Diet
5. Sleep
6. Social and Psychological Health
7. Vascular health (the health of your blood vessels and risk of heart disease/stroke)
8. Vision and hearing

**You will be asked to spend about 40 minutes per week on the program**. Each week you will receive an email notice alerting you to new program content. Information within the content modules will contain scientific evidence, specific recommendations for lifestyle changes (e.g. physical exercises to try at home, healthy recipes), and suggestions to improve behavior in everyday life and tips to make positive changes. Content format will include visual and auditory text, pictures, animations, quizzes, questions and interactive exercises.

**BHPro Lifestyle Questionnaires and Goal Setting:**

Approximately every three months, you will be asked to complete brief online lifestyle risk questionnaires. Questions will be related to the content in the program modules (e.g. sleep, diet, physical activity, cognition, social engagement, vision and hearing). This will allow you to receive feedback on your personal lifestyle risk factors. You will also be asked to set goals based on your risk factors and to re-visit and update your goals approximately every 3 months. You will be encouraged to focus on the content modules that are related to your risk profile.

**BHPro Program Evaluation:**

During the course of your 45-week participation in BHPro, we will be collecting data to assess how effective it is. Some of this data will be automatically collected from the program (e.g. number of chapters you complete and how many questionnaires you complete). At the beginning of the study, at approximately Month 6, and approximately Month 12 we will also ask you to spend about 30 minutes answering questions to evaluate the program including:

- Questions that will ask you about your current knowledge of Alzheimer’s Disease, your attitude and feelings about being screened for dementia, and your perceived ability to accomplish tasks and cope with difficulties
- Questions about your satisfaction with using the online program (e.g. do you like the website, is it easy to use, is the information easy to understand)

**Visit 3 (Month 3)**

You will receive a follow-up phone call from a study team member. They will ask you about your continued interest in the study, your satisfaction with BHPro, and address any questions you might have. The phone call will take about 45 minutes to 1 hour to complete.

You will also be asked to complete the following **study activities at home**:

- **Smartphone Memory Testing:** You will also receive a notice from the MyCogHealth app on your smartphone to complete another round of short daily tests of memory and thinking (5-10 minutes a day) on your smartphone for 7 days.
- **BHPro Lifestyle Questionnaires and Goal-Setting:** You will complete the brief online lifestyle risk questionnaires again and review the goals set within BHPro.

**Visit 4 (Month 6)**

You will receive a follow-up phone call from a study team member. They will ask you about your continued interest in the study, your satisfaction with BHPro, any changes in your health that have occurred since the last study visit, and address any questions you might have. The phone call will take about 45 minutes to 1 hour to complete.

You will also be asked to complete the following **study activities at home**:

- **Smartphone Memory Testing:** You will also receive a notice from the MyCogHealth app on your smartphone to complete another round of short daily tests of memory and thinking (5-10 minutes a day) on your smartphone for 7 days
- **Computer Memory Testing:** You will be asked to complete another round of brief tests of memory and thinking Cogniciti Brain Health Assessment) on your computer. This should take about 20 minutes to complete.
- **BHPro Lifestyle Questionnaires and Goal-Setting:** You will complete the brief online lifestyle risk questionnaires again and review the goals set within BHPro.
- **BHPro Program Evaluation Questionnaires:** You will complete brief online questionnaires about your satisfaction with using the online program (e.g. do you like the website, is it easy to use, is the information easy to understand).

**Visit 5 (Month 9)**

You will receive a follow-up phone call from a study team member. They will ask you about your continued interest in the study, your satisfaction with BHPro, and address any questions you might have. The phone call will take about 45 minutes to 1 hour to complete.

You will also be asked to complete the following **study activities at home**:

- **Smartphone Memory Testing:** You will also receive a notice from the MyCogHealth app on your smartphone to complete another round of short daily tests of memory and thinking (5-10 minutes a day) on your smartphone for 7 days
- **BHPro Lifestyle Questionnaires and Goal-Setting:** You will complete the brief online lifestyle risk questionnaires again and review the goals set within BHPro.

**Visit 6 (Month 12)**

At the end of your 45-week participation in BHPro, you will be asked to complete the following study activities over video-call with a study team member. The total approximate time to complete the Visit 6 study activities is 2.5 hours. The Visit 6 study activities include:

- **Medical History:** We will review any changes in health that you may have experienced since your last follow-up call.
- **Medications:** We will review your current medications.
- **Weight:** We will ask you to tell us your approximate weight.
- **Memory Testing:** You will complete brief tests on your memory and thinking. Most of these tests will involve question and answer format but some may involve paper and pencil. You will be able to skip any questions that you are uncomfortable answering.
- Your study partner (if you have one) will also be asked questions about your memory and thinking skills.
- **Neurological Exam**: A study doctor will evaluate you and complete a brief neurological exam over video-call. The study doctor or research staff will let you know what will happen during the exam.

You will also be asked to complete the following **study activities at home**:

- **Smartphone Memory Testing:** You will receive a notice from the MyCogHealth app on your smartphone to complete another round of short daily tests of memory and thinking (5-10 minutes a day) on your smartphone for 7 days
- **Computer Memory Testing:** You will be asked to complete another round of brief tests of memory and thinking (Cogniciti Brain Health Assessment) on your computer. This should take about 20 minutes to complete.
- **Physical Activity Monitoring:** Wear the wristwatch-like (actigraph) device continuously for another 14 days and mail it back to the study site using a pre-addressed, stamped envelope that we will be given to you by a member of the study staff. You will also be asked to keep a sleep and work diary for these 14 days.
- **Sleep Recording**: Wear the EEG sleep headband and use study smartphone to record your sleep activity for another 3 nights in a row and mail it back to the study site using the pre-addressed, stamped envelope that will be given to you by a member of the study staff. You will also be asked to fill out a survey to collect your feedback about the sleep headband after wearing it for the 3 nights.
- **BHPro Lifestyle Questionnaires and Goal-Setting:** You will complete the brief online lifestyle risk questionnaires again and review the goals set within BHPro.
- **BHPro Program Evaluation Questionnaires:** You will complete brief online questionnaires about your satisfaction with using the online program (e.g. do you like the website, is it easy to use, is the information easy to understand).

**Recording Video-Call Study Visits**

The memory testing portion of your video-call study visits (visits 1, 2, and 6) will be recorded to help the study team member, who is conducting your study visit, accurately score your study tests and make sure that all of your study data is complete. Video recordings will only be viewed by the study team member who is conducting your study visit and/or the supervising neuropsychologist and will be deleted once your data has been entered into the study database (but no longer than 60 days after the recording). The audio files from the recordings will be uploaded to the study database. The recordings will be labelled with a study code and will not have any identifying information attached to them. The audio-recordings will only be accessible to study team members and will be deleted from the study database after data analysis is complete. A copy of the audio-recording will also be kept on an external hard drive at the site of your assessment that will not be connected to the internet and kept in a locked cabinet. The audio-recording will be kept for at least XX years at the local study site.

**Genetic Research**

During this study, a saliva sample will be collected from you for genetic research. The sample will only be used for the research described in this consent form, and will not be used for any other purposes. Your sample will be stored by code number and no identifying information will be included with it.

Your sample will be sent to the Clinical Genomics Centre in the Mount Sinai Hospital, in Toronto, Ontario and will be processed under the guidance of Dr. Kathy Siminovitch. The sample you send in will be labelled with your study ID, and will not have any identifying information. The results of these tests will be maintained in scientific databases for this research study. These results are important only for research - not for helping to care for you. For this reason, the results will not be released to you or your family. No information regarding your genetic research will be entered into your regular medical record. Data from your tests will not be revealed to other sites that are participating in the clinical study, family members, insurance companies, employers, or other individuals or organizations. Your sample will be stored according to standard lab procedures for the duration of study and until all planned analyses are complete, after which it will be safely destroyed.

Although the study researchers will have access to coded individual data, any information gained from this research will be reported in publications in an anonymous summary form. Data will be stored in a locked file, and in a computer with restricted access. Any information that could be used to potentially identify you in the computer will be stored in a separate file and encrypted. Only the researchers and their research assistants will have access to the original research data. The genetic information that is obtained from the analysis of your saliva sample may be linked to other data collected about you during this study, or other CCNA studies that you may have participated in. This information may also be shared with other researchers as described in the section below “Will this research data be shared?”

When you donate your saliva for genetic testing or research, you are sharing genetic information, not only about yourself, but also about biological (blood) relatives who share your genes or DNA.  The risk of your information being accidentally released in this study is estimated to be low. A Federal (Canada-wide) law now prohibits anyone such as an employer or an insurer from requiring you to disclose the results of a genetic test or to take a genetic test as condition of providing services.  In addition, discrimination against individuals based upon genetic characteristics is now prohibited by the Canadian Human Rights Act.

**WHAT ARE THE RISKS OF THE STUDY?**

Being in this study involves some risk to you. You should discuss the risk of being in this study with the study staff. Risks and side effects related to your participation in this study are outlined below.

**Risks of Memory Testing and Mood Assessments**

Repeated testing and questions about your mood and mental status may be slightly frustrating or produce fatigue and boredom. Some questions may trigger distressing feelings or memories. If you feel distressed while completing the online questions or testing, take a break for a little while. If, after some time, you do not feel you can complete the questions, you can contact the study staff at [Insert Site Phone Number]. They will consult with you about proceeding with the rest of the evaluations and respect your decision.

**Risks of Physical Activity and Sleep Recordings**

There are no known medical risks to you from using the actigraph or EEG sleep headband to record your sleep and daily activity. However, you may experience side effects from participating in these procedures. Some side effects are known and are listed below, but there may be other side effects that are not expected.

- There may be some minimal discomfort from wearing the wristwatch-like actigraph device.
- There may be some minor disturbance of your usual sleep on the nights that you are wearing the EEG headband.
- You may learn something about your sleep that you were unaware of, which could cause some anxiety or discomfort. If there is a significant medical problem identified, appropriate care will be arranged for you.
- You will be told about any new information that might reasonably affect your willingness to continue to participate in this study as soon as the information becomes available to the study staff.

If you experience these or any other side effects from the sleep and physical activity recording devices, you should contact the study staff at [Insert Site Phone Number] about any side effects or study-related injuries that you experience.

**Risks to Privacy**

We will gather data from you from multiple sources including the BHPro website, Cogniciti website, the MyCogHealth app on your smartphone, and the devices that you will wear to record your physical activity and sleep. There is a risk to your privacy whenever you use an app or website. If there is a data breach, someone could see or use the data we have about you. We believe this risk is very small but it is not zero.

**Risks of Genetic Testing**

One of the risks associated with this research project relates to the disclosure of the results or the disclosure of your participation to third parties. For example, the information could potentially be used against participants if it were revealed to insurance companies or potential employers. You will not get the results of the genetic portion of the study and the results will not be included in your medical record. The researchers have adopted strict privacy and confidentiality procedures for maintaining your genetic information, as described in this consent form.

**WHAT ARE THE POTENTIAL BENEFITS OF PARTICIPATING?**

There is no guarantee that you will personally benefit by participating in this research study. Participating in a web-based educational program may lead to increased knowledge and engagement regarding ways to promote your brain health, and possible positive reduction in your dementia risk profile, but such improvement cannot be known with any certainty. This study is expected to benefit the general community in the future by promoting public interest, literacy, and engagement in lifestyle interventions for brain health and dementia prevention through circulation of BHPro to the broader public. There is a possibility that the study website, app, devices, or results of this research may be used for commercial and /or intellectual property purposes (ie. patents) in the future. The researchers would claim sole ownership of the commercial and/or intellectual property developed from the results of this research.

**HOW WILL MY RESEARCH DATA BE USED AND STORED?**

All data collected in this study will be stored in the Longitudinal Online Research and Imaging System (LORIS), a restricted access database at McGill University in Montreal that meets international security and safety standards. All electronic data collected from websites, smartphones and physical activity and sleep recording devices (actigraph devices, EEG headbands, iPods, Cogniciti) will be saved on secure servers before being transferred to the LORIS database. The server for the EEG data is located in the USA. A number of safeguards are in place to keep your information confidential. In particular:

- Personal identifiers will be removed (i.e. name, birth month and year, etc.);
- Your personal details will be kept separate from your research data;
- Your data will be coded (attached to a random series of numbers) which will be how it is identified within the study
- Strict security measures will prevent unauthorized access or misuse.

Any information that could be used to potentially identify you in the computer, will be stored in a separate file and will be encrypted. Only the study researchers and their research team members will have access to the original study data. These safeguards make it difficult to know which personal information came from you or any other participant. However, we cannot guarantee that you will never be re-identified, meaning that even though we have taken every precaution to collect only general, non-identifying information from you for the dataset, and to store it securely, there is a very small chance that sophisticated technology could be used to re-identify the data as belonging to you, in the event of a privacy breach. In the event of a problem with privacy, the site study investigator or his/her delegate will notify you immediately. Only coded data, which does not include anything that might directly identify you, will be shared for research purposes.

**Will My Information Be Kept Private/Confidential?**

Your confidentiality will be respected. However, research records and health or other source records identifying you may be inspected in the presence of the Investigator or his or her designate by representatives of the [Insert Name of Site REB] or by the study Sponsor, CCNA, for the purpose of monitoring the research. No information or records that disclose your identity will be published without your consent, nor will any information or records that disclose your identity be removed or released without your consent unless required by law.

You will be assigned a unique study number as a participant in this study. This number will not include any personal information that could identify you (e.g., it will not include your Personal Health Number, SIN, or your initials, etc.). Only this number will be used on any research-related information collected about you during the course of this study, so that your identity will be kept confidential. Information that contains your identity will remain only with the Principal Investigator and/or designate. The list that matches your name to the unique study number that is used on your research-related information will not be removed or released without your consent unless required by law.

Your name, participant number, address, and contact information will be shared with study staff at Sunnybrook Research Institute (SRI, University of Toronto, Ontario) via encrypted email. Study staff at SRI will mail study supplies to your home and will also provide you with instructions on how to use the wearable devices (e.g. sleep headband, actigraph, iPod). Your name, participant number, and contact information may also be shared with study staff at the CogTech Group at the University of Victoria via encrypted email. Study staff at the CogTech group may contact you if you need technical support or help using the MyCogHealth app on your smartphone.

Your rights to privacy are legally protected by federal and provincial laws that require safeguards to ensure that your privacy is respected. You also have the legal right of access to the information about you that has been provided to the sponsor and, if need be, an opportunity to correct any errors in this information. Further details about these laws are available on request to your study doctor.

Any study related data, sent outside of Canadian borders may increase the risk of disclosure of information because the laws in those countries, dealing with protection of information may not be as strict as in Canada. However, all study related data, that might be transferred outside of Canada will be coded (this means it will not contain your name or personal identifying information) before leaving the study site. By signing this consent form, you are consenting to the transfer of your information, to organizations located outside of Canada. All servers are located in Canada, with the exception of the cloud EEG server, which is located in the USA.

**Will This Research Data Be Shared?**

Data sharing are important for further translation of research results into knowledge, products, and procedures to improve human health. Data from this research study, once they have had all identifying information removed, may be shared with CCNA researchers. If you agree to participate in another CCNA sponsored study, you may be asked for permission to have the data collected in this CTU study to be used as part of that study. Data may also be shared with other researchers around the world and used in future research projects. In order to access the data generated in this study, researchers must agree to abide by the CCNA Publication and Data Access Committee policy, a document prepared by the CCNA Publication and Data Access Committee (PDAC), which can be downloaded at [www.ccna-ccnv.ca](http://www.ccna-ccnv.ca). Researchers will need to submit a specific request and receive approval from the PDAC before getting access to the study data. They will only be granted access to data related to the project outlined. These projects can take place in universities, hospitals, non-profit groups, companies, and/or government laboratories. All researchers must respect the laws and ethical guidelines for biomedical research.

**WHAT ELSE DO I NEED TO KNOW BEFORE PARTICIPATING?**

**Can I Be Asked to Leave the Study?**

Participation in this research study is entirely voluntary. You have the right to stop participating in this study at any time. In addition, the study investigator can stop your participation at any time without your consent for any of the following reasons:

- If it appears to be medically harmful to you;
- If you fail to follow directions for participating in the study;
- If it is discovered that you do not meet the study requirements;
- If the study is canceled

If you stop participating in the BHPro intervention for any reason, you may have the opportunity to complete further study visits up until the end of the study. If you do not wish to complete further study visits, you will be asked to allow a study team member to contact you by phone and/or video call for a final study assessment. This final assessment will include all of the procedures normally performed at Visit 6 (Month 12). It is important for the research to have these final procedures completed. Your participation in the study may be stopped by the study investigator if you experience a medical condition that makes it unsafe for you to continue in the study.

**What Happens If I Decide to Withdraw My Consent to Participate?**

You may withdraw from this study at any time without giving reasons. If you choose to enter the study and then decide to withdraw at a later time, the study team will have a discussion with you about what will happen to the information about you and/or your samples already collected. You have the right to request the destruction of your information and/or samples collected during the study, or you may choose to leave the study and allow the investigators to keep the information already collected about you until that point.

If you choose to have the data collected about you destroyed, this request will be respected to the extent possible. Please note however that there may be exceptions where the data and/or samples will not be able to be withdrawn for example where the data and/or sample is no longer identifiable (meaning it cannot be linked in any way back to your identity) or where the data has been merged with other data. If you would like to request the withdrawal of your data and/or samples, please let your study doctor know. If your participation in this study includes enrolling in any optional studies, or long term follow-up, you will be asked whether you wish to withdraw from these as well

**Medically Significant Findings**

Most tests done in research studies are only for research and have no clear meaning for health care.

Occasionally, an unexpected finding comes up in the course of assessing a participant that may require further medical attention. For instance, learning that a participant has sleep apnea, or depression, which should be treated. In order to participate in this study, you must identify a family doctor or other healthcare provider and provide permission for us to let your provider know that you are a participant in this study. If the study investigator determines that any of their findings from your study examinations are medically significant or might change your treatment, they will share these medical findings with your healthcare provider so that s/he can review them with you and discuss potential treatment or follow-up options. The finding and its follow-up will be documented in your research file and its outcome will be monitored until it has been resolved or as long as you remain in the study.

**What Other Choices Are There?**

This is not a treatment study. Your alternative is to not participate in this study.

**What will the Study Cost me?**

There will be no costs to you for participation in this study.

**Remuneration**

Procedures performed for the purposes of this study will be provided at no charge to you. You will be compensated a total of $100 for your time and effort in this study. You will be compensated $50 following your Visit 2 (Baseline) phone/video-call and an additional $50 at the end of your Visit 6 (Month 12) phone/video-call or upon your early withdrawal from the study.

**What Happens If Something Goes Wrong?**

Should you suffer harm of any kind following any procedure related to the research study, you will receive the appropriate care and services as required by your state of health. There will be no other form of compensation to you if you are injured.

You should inform the healthcare professional treating you that you are participating in this study. If you tell the study staff that you think you have been injured then they will help you get the care you need.

By signing this form, you do not give up any of your legal rights and you do not release the study doctor, participating institutions, or anyone else from their legal and professional duties. If you become ill or physically injured as a result of participation in this study, medical treatment will be provided at no additional cost to you.

**If I Have Questions About the Study Procedures During My Participation, Who Should I Speak to?**

If you have any questions or desire further information about this study before or during participation, or if you experience any adverse effects, you can contact [Insert Name of Site PI], the Principal Investigator at [(XXX) XXX-XXXX] or [Insert Site Coordinator Name], the study coordinator, at [(XXX) XXX-XXXX].

**Who Do I Contact If I Have Questions or Concerns About My Rights as a Research Participant?**

If you have any concerns or complaints about your rights as a research participant and/or your experiences while participating in this study, contact the [Insert non-study contact e.g. Manager of REB] at [(XXX) XXX-XXXX ext: XXXXX].

**AFTER THE STUDY IS FINISHED:**

A description of this clinical trial will be available on http://www.ClinicalTrials.gov. This website will not include information that can identify you.The website will include a summary of the results and publications resulting from this research. You can search this website at any time.

**USING EMAIL TO COMMUNICATE**

We are asking to collect your email address because we will be setting up remote visits with you via video-call and will need to send and receive materials over email to facilitate the visit. Before you provide your consent, please carefully consider whether this email account is secure, whether other people have access to it or whether you have concerns about the security of any information sent to this account. We will only send your personal information to the email address you have provided to us, and all of the information which you provide to us will be kept confidential by the research team. The security of information sent over the internet can also not be guaranteed. The email communications we exchange with you may contain some of your personal information (your name, health information, etc.). By providing your email address, you are voluntarily providing your consent for the study team to communicate with you using your email account.

**PARTICIPANT CONSENT**

The Canadian Therapeutic Platform Trial for Multidomain Interventions to Prevent Dementia: Brain Health Support Program Intervention (CAN-THUMBS UP or CTU)

My signature on this consent form means:

- I have read and understood the information in this consent form.
- I have been able to ask questions and have had satisfactory responses to my questions.
- I understand that my participation in this study is voluntary.
- I understand that I am completely free at any time to refuse to participate or to withdraw from this study at any time, and that this will not change the quality of care that I receive.
- I understand that I am not waiving any of my legal rights as a result of signing this consent form.
- I understand that my data will be stored and used for the research described above.
- I understand that my saliva sample will be securely stored and used for research purposes only. This includes my DNA. Information that researchers learn by studying my samples will be stored in secured study databases.
- I authorize access to my health records and samples as described in this consent form.
- I authorize the study team to notify my family doctor or other designated healthcare provider that I am participating in this study.
- I authorize the study team to forward any abnormal results to my family doctor or other designated healthcare provider.
- I understand that my name, study number, address, and contact information will be shared with study staff at Sunnybrook Research Institute (SRI, University of Toronto). Study staff at SRI will mail study supplies to my home and will also provide me with instructions on how to use the wearable devices (e.g. sleep headband and actigraph).
- I understand that my name, study number, and contact information may be shared with study staff at the CogTech Group at the University of Victoria. If I need technical assistance or help using the MyCogHealth application on my phone, study staff from the CogTech Group may contact me.
- I understand that some of my study visits will be recorded. Video-recordings will be permanently deleted within 60 days from the recording. Audio-recordings will have identifying information removed and will be securely stored in the study database. Recordings will not be shared with anyone outside of the study team.
- I understand that there is no guarantee that this study will provide any benefits to me.
- I understand that as a member of the CAN-THUMBS UP: BHSP study group, I may become eligible to further consent and participate in future studies carried out by CAN-THUMBS UP. I give CAN-THUMBS UP permission to contact me about future opportunities.
- I will receive a signed and dated copy of this consent form for my own records.

I have read this consent form and completed the e-consent process. I understand the information in this form. All of my questions have been answered. I freely and willingly choose to take part in the Canadian Therapeutic Platform Trial for Multidomain Interventions to Prevent Dementia (CAN-THUMBS UP or CTU): Brain Health Support Program Intervention (BHSP)

**I AGREE I DISAGREE**

**
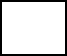
**

**I consent to participate in this study.**

Participant’s Signature Printed Name Date

_______

Signature of Person Printed name Study Role Date

Obtaining Consent

My signature may have been added at a later date, as I may not have been present at the time the participant’s signature was obtained.

**Study Partner** (Optional)

If you have someone close to you (spouse, friend, relative), with whom you have contact on a weekly basis, they may also be invited to participate in this study as a “Study Partner”.

Their involvement will include answering questions about your memory and everyday functioning during the Visit 1,2, and 6 phone/video-calls. If you do not have a Study Partner, you will still be able to participate in this study.

**Do you have a Study Partner available that you would like to be involved in this study?**

If you select “yes”, a study team member will contact you for your Study Partner name and contact information. A study team member will contact your potential Study Partner and provide them with a separate Study Partner Information and Consent form that outlines the study and what their involvement would be.

**NO**

**YES**
