# Supplementary material for: Protocol for the Brain Health Support Program Study of the Canadian Therapeutic Platform Trial for Multidomain Interventions to Prevent Dementia (CAN-THUMBS UP): A Prospective 12-Month Intervention Study
Source: J Prev Alzheimers Dis. 2023 Jun 15:1–11. Online ahead of print. doi: 10.14283/jpad.2023.65 (PMC10258470; doi:10.14283/jpad.2023.65)
Supplement: Supplementary file 2 — Supplementary material, approximately 13.3 KB. [file 42414_2023_227_MOESM2_ESM.docx]

**Appendix B: Saliva Sample for Genomics Testing**

A saliva sample collection kit (OG-500 kit, DNA Genotek, Kanata, Ontario) is sent to each participant for extraction of DNA. Study staff at Sunnybrook Research Institute are responsible for mailing the kits to the participant’s home. The at-home kit includes detailed instructions on how to collect the sample and a study team member provides guidance to the participant on how to properly collect and ship the sample during the Baseline video-call visit. A total of 2 mls of saliva are collected by passive drool in 1 tube. Participants are instructed not to eat, drink, smoke, or chew gum for 30 minutes prior to giving the sample. The samples are collected, handled, stored and shipped according to established standard operating procedures. Instructions for collection, handling and shipping of saliva samples for DNA testing are also provided in the Study Procedures Manual.

De-identified samples are sent to the Clinical Genomics Centre in the Mount Sinai Hospital, 600 University Ave, Toronto, ON M5G 1X5, Canada and processed under the guidance of Dr. Kathy Siminovitch. Samples will be stored for the duration of the study and until all planned analyses are complete, after which they will be properly destroyed according to established lab procedures.

A Polygenic Hazard Score (PHS) will be derived from a planned panel of single nucleotide polymorphisms that describes age of onset and AD risk. Both raw and processed data will be posted to the LORIS server.
